# Death and Nationalism's Moral Imperative: The Battle for Britain, Industry and the ‘Left Behind’

**DOI:** 10.1111/1468-4446.13209

**Published:** 2025-03-24

**Authors:** Bethan Harries

**Affiliations:** ^1^ Sociology Newcastle University Newcastle upon Tyne UK

## Abstract

This paper is concerned with how nationalism is convened and condensed in this moment by exploring the function of loss and death and their centrality to nationalism's articulation. The discussion attempts to make sense of how death possesses an ideological currency that wields an alluring quality and equips nationalism with a moral imperative. This focus is stimulated by the abundant rhetoric which draws on real, mythologised and metaphorical deaths, to imply the ‘killing off’ of our communities, our industrial heartlands, our values, our nation, etc., and which has been a perennial feature of English nationalisms but which has intensified since the Brexit campaigns, their enduring legacy and the general move to the right. The racialised dimensions of these arguments are recognised as vital to reveal the close imbrication of the narration of race, class and nation and the various claims made through their articulation with death, including how this underpins who is worth saving and not. Indeed, the key aim of the paper is to demonstrate nationalism's capacity to simultaneously produce the moral imperative for sacrifice for authentic (often white working class) subjects and the brutal abandonment of racialised ‘others’ for the sake of the longevity of the nation. In short, it seeks to better understand how lives are said to matter and not, especially in times of economic hardship. I propose that the integrity of the nation claims to be reliant on the sacrifices of the, implicitly white working‐class ‘left behind’ via austerity, Brexit and beyond, but that this is simultaneously contingent on the brutal abandonment of racialised others.

## 1 Introduction

This paper is concerned with how nationalism is convened and condensed in this moment by exploring the function of loss and death and their centrality to nationalism's articulation. The discussion attempts to make sense of how death possesses an ideological currency that wields an alluring quality and equips nationalism with a moral imperative. This focus is stimulated by the abundant rhetoric which draws on real, mythologised and metaphorical deaths, to imply the ‘killing off’ of our communities, our industrial heartlands, our values, our nation, etc., and which has been a perennial feature of English nationalisms but which has intensified since the Brexit campaigns, their enduring legacy and the general move to the right. The racialised dimensions of these arguments are recognised as vital to reveal the close imbrication of the narration of race, class and nation (see e.g., Rhodes et al. 2019; Bhambra 2017; Mondon and Winter 2018; Virdee and McGeever 2018; Patel and Connelly 2019) and the various claims made through their articulation with death, including how this underpins who is worth saving and not. Indeed, the key aim of the paper is to demonstrate nationalism's capacity to simultaneously produce the moral imperative for sacrifice of authentic (often white working class) subjects and the brutal abandonment of racialised ‘others’ for the sake of the longevity of the nation. In short, it seeks to better understand how lives are said to matter and not, especially in times of economic hardship. I propose that the integrity of the nation claims to be reliant on the sacrifices of the, implicitly white working‐class ‘left behind’ via austerity, Brexit and beyond, but that this is simultaneously contingent on the brutal abandonment of racialised others.

From the outset, I suggest that Sociology has typically overlooked the nation except where it is identified as a means of bounded inclusion/exclusion and as such the premises that the nation relies upon in its very construction often remain overlooked/unchallenged. And yet, there is a pressing need to better understand how nationalism is made to work so successfully in the current conjuncture and to *undo* the underpinning logics of propositions and claims made in the production of the nation. To attend to this need, the paper focuses on the centrality of industry to the conjuring of Britain and Britishness and subsequently proposes that the loss of industry (notably not jobs) has become one of the most powerful tools in the arsenal of contemporary nationalism. In doing so, the discussion highlights the ways in which the industrial past (and what is said to have been lost) is so heavily relied upon as a means to reinforce the idea of the nation and reconfigure its future. However, it also draws attention to the ways in which a kind of ‘post‐industrial’ melancholia produces the moral justification for specific forms of sacrifice and abandonment.

The first two sections of the paper set up the framework for the discussion and the underpinning idea that the notion of death is key to articulations of death and nationalism. Within this, I take as a starting point that the recent reliance on ‘English post‐industrial left behindness’ (and its various synonyms) is a useful position to explore within this because it is harnessed by a broad political spectrum to apparently achieve different ends and therefore helps to reveal the force of nationalism that traverses ideological terrains through the moral imperative indicated by the need to attend to the very survival of the nation. Drawing on Gilroy's work on melancholia, the paper introduces the idea of post‐industrial melancholia to indicate the contradictions inherent in the mythology of success in relation to Britain's industrial story and the real experience of decline, which rely on the concealment of the terms on which the nation's grief for industry (and implicitly empire) is contingent.

The remainder of the paper draws on research that was carried out in the former shipbuilding town of Wallsend in North Tyneside during the ‘Brexit period’ in order to think carefully about how nationalism (underpinned by race) is put to work hand in glove with anxieties over the death of industry. It takes a particular interest in the notion of the deindustrialised ‘left behind’, which has emerged quite forcefully in the Brexit period, in order to understand the significance of the ways in which death and loss can act as emissaries for contemporary nationalism. It does this by placing an analysis of the current conjuncture in conversation with a longer history of Wallsend, including via conversations with residents that aimed to stimulate people's reflections about the future ‘after’ Brexit, but which elicited contradictory narratives of history and longing and the call for sacrifice and abandonment. The intention of this discussion is partly to provoke close reflection on why the moniker of the ‘left behind’ has come to matter in this conjuncture, when it is put to use as a national motif of loss. But also how the motif of the post‐industrial ‘left behind’ works in a place such as Wallsend and can illustrate how death and loss carries with it an underlying message that has much further reach, to imply that the nation and ‘the people’ are at risk and are thus justified, and morally obligated even, in their defence of *it* and their grieving for *it*, as if it is a singular and fixed entity that is theirs to own and protect.

## 2 Death, Loss and the Moral Imperative of Nationalism

The notion of death is fundamental to articulations of nation and nationalism. The story of the nation facilitates a sort of social immortality in which a mythical ‘we’ can live on as members of that community/nation even after their own demise. Nations are imagined to be ‘communities’ that imply such a deep sense of comradeship that people are willing to die for them to insure that immortality (Anderson 1983). But, more intrinsic to nationalism is the urge to assuage anything or anyone that might be constructed as a threat to the bounded life of the nation or, more specifically, the illusion of the nation as a singular unified reality. It is no surprise then that in the current conjuncture, where ‘nationalism is everywhere’ (Valluvan 2019), death and loss feature so prominently in its articulation and in the ciphers through which it is delivered. And, whilst this might be most viscerally felt in the mainstreaming of right wing ideologies (think, e.g., of the ‘great replacement’ theory), the utter disregard for the tragic deaths of thousands of people seeking refuge, or when thinking about those who were sacrificed to enable ‘eating out to help out’, it is also evident in liberal anxieties about *numbers* of migrants, who has access to ‘national’ resources (even where those resources are privatised), who deserves care, especially in later life, and the right and wrong ways we should remember and teach ‘our’ history and whose lives are worth remembering or not. In short, anxieties that some ‘thing’, or some essence of ‘nationalness’ is being lost, or is under attack, and is at risk of expiration is articulated and meted out into the most mundane spaces, conversations and experiences and traverse different social, political, and ideological terrains. These anxieties do considerable work and this reaches well beyond who can be said to belong and who cannot. They express what ‘needs to be done’, what ‘*must* be done’ to secure the nation's longevity by articulating who and what is worthy of saving, caring for and about.

Nationalism proffers a collective sense of meaning even whilst people's lives are decimated for the ‘collective good’. They place a value on people's lives which lays bare stark inequalities whilst simultaneously attempting to conceal them or, increasingly as is the case, to justify them. For nationalism it does not matter whether the sense of loss on which it relies is based on real, metaphorical or mythologised death. What matters is *how* that grief and loss is made to signify import for the nation, including who is worthy of our collective grief and who is said to be responsible for it; whose deaths are worth commemorating and whose we should begrudgingly or scornfully accept, and even let happen. Austerity measures, put simply, led to the deaths and decline of many people's lives and the evisceration of whole neighbourhoods and regions, and were justified via narratives of sacrifice and their necessity to secure the nation's longevity. Indeed, David Cameron, then PM, crystalised this in his speech to conference in 2013 when he thanked, ‘the British people’ for their ‘sacrifice’, without grieving, or even recognising, those lives lost for, ‘never giving up. Working those extra hours. Coping with those necessary cuts’, in the same breath as thanking British soldiers in Afghanistan (see also Nail 2013). Posited against this is the contingent logic that racialised minorities can ‘justifiably’ be blamed for threatening the life of the nation by taking away its resources and polluting its culture and social life whilst their own lives are forsaken. Indeed, vast racial inequalities are ignored and concealed, including those that reveal brutal disparities in maternal deaths, deaths in custody, excess deaths during the pandemic, and so on (see Williams et al. 2023; Athwal and Burnett 2014; Clarke 2021; Murji and Picker 2021; Nazroo and Becares 2020; Knight et al. 2020; Dayo et al. 2023). Vital scholarship and activism has repeatedly deconstructed and disrupted the myths and misrepresentations that are forged amongst the multiple and connected nefarious projections made around loss and nationhood, or more particularly who and what is to blame for the nation's ‘loss’ and threats to its (cultural, economic and social) demise. This is the case via contestations over: national identity (Habermas 2001), a politics for recognition and multiculturalism (Hall 2000), or who is deserving and who is not (Shilliam 2018). However, what often remains unquestioned/unchallenged, in Sociology at least, are some of the underlying premises on which the nation is imagined and uttered into existence.

Sociology has tended to frequently ignore the nation and those premises that are not only said to define ‘Great’ Britain but also place it full and centre on the global stage. Britain's role as ‘great’ architect of Empire proves a growing exception here (an indicative example might include, Evans and Williams 2024; Bhattacharyya et al. 2021; Koram 2023; El‐Enany 2021), but I am thinking too about the premise of Britain as the ‘birthplace’ of the industrial revolution, victorious ally in WW2, and loyal to and proud of its royal family and the commonwealth. Yet, it is these modern premises that conjure a particular enduring imaginary more successfully than any calls to primordial heritage. The right has learnt this too hence in the current conjuncture we no longer hear the likes of the British National Party's urge to maintain a genetically white population and prevent the extinction of ‘unique human genotypes’ as Nick Griffin did on BBC's Question Time in 2009. Instead, we are more likely to repeatedly hear of the need to protect borders, ‘sovereignty’ (often ambiguously defined to stand in for borders), as well as the glorification of Britain's indomitable industrial heritage and global imperial might (for further discussion in relation to this idea see e.g., Virdee and McGeever 2023; Bhambra 2007). In other words, as Valluvan notes, nationalists also ‘might understand the nation to have emerged through a particular history’ (Valluvan 2019, 32) but can nevertheless prioritise the unique nature, strengths and qualities of the nation they seek to uphold (Valluvan 2019). Consequently, it is these modern premises which form the basis on which anxieties about loss can successfully be made to work, foregrounding as they do a glorification of the past which does as much work to distract/conceal wrongdoings, but also justify a lack of care about the lives and deaths of others. It is at this point that I want to turn to briefly consider the role of industry as part of a broader nationalist project and how this articulates with loss and the signification of ‘left behindness’.

## 3 Post‐Industrial Melancholia and the ‘Left Behind’

Even when Britain is in economic turmoil, and its GDP and its manufacturing output is in decline, we nevertheless relentlessly hear an echo of Great British industry. In the current boom in nationalist driven programming TV shows make somewhat tragic attempts to resuscitate pride in British industry, offering a lacklustre lament of industrial innovation alongside artisanal entrepreneurialism. The BBC's ‘Inside the Factory’, for example, gives us Gregg Wallace, who is perhaps better known for touting the benefits of high‐end cuisine in Masterchef, trekking around English provincial towns proudly showing us the inner workings of the likes of a processed cheese factory in Gateshead, or a sausage factory in Thirsk. This is neatly posited against the plethora of TV shows prefixed by ‘The Great…’ or ‘The Great British…’ which showcase artisanal skills (baking, carpentry, pottery, sewing, interior design and so on) that have ‘farmer's market’ entrepreneurial potential. These kind of cosy appeals to collective skills and outputs do not displace the idea of Britain as a key contender on the global stage. Rather, they proffer us a vision of a future in which factories still loom and ‘the people’ of the nation remain highly skilled, innovative and, crucially, entrepreneurial. Underlying this output too is the incitement to *buy British* as a means to support the national economy and its entrepreneurial spirit and, although this is a by‐product to a broader project, it appeals to a common‐sensical moralising atmosphere which reaches across a broad political spectrum by virtue of its apparently innocuous message that we *must* save the nation. Alongside this are renewed calls that ‘we’ must harness our entrepreneurial potential, an idea which is often explored in relation to neoliberalisation but nevertheless has its roots in the ‘colonial enterprise’ (Calhoun 2006; Bhambra 2007; O'Hearn 1994; Balibar and Wallerstein 1991) that continues to underpin the contemporary national imaginary, cast as it is as the means through which to resuscitate the nation, via calls for ‘entrepreneurial courage’ (McKeon june 2024) etc.

As with so many of the ingredients of nationalism the contradiction of mythologised success (e.g., of industry) and the real experience of decline does not have to matter. It is important to note then how the success of Britain's industrial dominance is not solely mythologised in a way that denies the contribution of the colonised, and the inequalities it inevitably produces (O'Hearn 1994; Bhambra 2007), but also mythologised to assume an evenness to the progression of that dominance. This is important because it is relied upon so heavily to tell the story of the nation. Multiple interpretations of the size, impact and evenness of the industrial revolution are available within historical analysis (see e.g., Hudson 1992; Hobsbawm 1999) and yet, the cultural production and the signification of the industrial revolution and industry to the narrative of the nation very much resembles that interpretation that persisted in the early nineteenth century through which the progress of Great Britain should be considered with ‘wonder and astonishment’ (Colquohon 1814 reprinted in Hudson 1992, 10). Indeed, the narrative of the nation that underpins the story of Great Britain is one that emphasises the role of Britain as pioneer, as world leading and as teacher and civilising force, in respect of showing the way for ‘less developed’ economies to follow. The unevenness of the impact of industry, including global and regional disparities and widening inequalities and the devastating impact on living conditions of workers, are well documented (Hobsbawm 1999) but of course do not fit the triumphant narrative and thus remain silenced.

Gilroy (2004) illuminates how it is imaginable to grieve the nation's losses without acknowledging that on which they are said to be contingent. Britain's imperial and colonial history remains unspoken in any sensible or profound way even whilst it continues to shape political and economic global relations. Yet, this history relentlessly and conveniently floats to the surface to underpin national celebrations, glorifications and commemorations as a symbolic gesture towards the nation's ‘great’ past (Gilroy 1992). As such, this brutal history remains trivialised and unresolved sitting inside a school curriculum that disavows its significance and yet also lists understanding right and wrong as a fundamental British value. Indeed, it is this struggle to reconcile Britain's identity as force of good and evil that propagates Britain's melancholic attachment to its past (Gilroy 2004). And it is precisely these reconfigurations of the past, that disavow the elements that are cumbersome to acknowledge, that enables nationalism to flourish based on a decapitated and romantic notion of some place that was once great and can be great again. For example, Virdee and McGeever (2018) detail how, much of the rhetoric that circulated during the Brexit period and what led to it, was underpinned by the image of Britain's imperial might and power as key proponent of global capitalism and imperialism, whilst occluding ‘the corrosive legacies of colonialism and racism’ (Virdee and McGeever 2018, 1803). However, they also highlight how, simultaneously, the Leave EU campaign relied on a discursive regime that emphasised a ‘more insular Powellite narrative of retreating from a globalising world that is no longer recognisably “British” (Virdee and McGeever 2018)’. Whilst these two distinct narratives appear on the surface to outwit each other, one locus where they converged was in their appeal to the nation's post‐industrial nostalgia and what became known as the ‘left behind’. On the one hand they kindled the return to a time where core industries, such as coal, shipbuilding, metals, machine production, etc. dominated the global market and were enmeshed in the financing of British imperial projects. On the other, they fed the autosuggested narrative that these *British* industries have been lost/taken away and usurped by other less deserving players. A well‐embedded racist discourse facilitated the transposition of this lament about industry in the same way that racialised minorities have been blamed for taking away jobs in areas with high unemployment even in those with low ethnic diversity, that is they appeal to a nationalist common‐sensical narrative of survival, irrespective of any sort of truth. It is important to note that whilst, contrary to early speculation, this vaguely identified group of ‘left behind’ voters did not form the majority of Leave voters (Dorling 2018), it was rather what the left‐behind narrative came to signify that matters in terms of the narration of nation.

## 4 Wallsend

In 2019, colleagues and I were awarded a grant under the ESRC's ‘governance after Brexit’ scheme. The broad aim was to explore how people living in de‐industrialised areas that had become labelled as ‘left behind’ envisaged the future after Brexit. Brexit was not defined specifically by one particular ‘epochal’ moment—that is not the referendum, not the campaign, but rather some ongoing ‘thing’ that has a ‘liquefied temporality’ and that could potentially be used to stand in as useful cipher to understand the current conjuncture. As it happened, during the lifecourse of the project, Brexit did not come to its completion and the events we had planned for the 29 March 2019, or ‘B‐Day’ as we referred to the original date of ‘exit’ and end of Article 50 period, were hastily reorientated. Nevertheless, the project maintained its focus throughout on how people were grappling with the future, which of course inevitably entailed tracing through an assemblage of the past to construct some sort of projection.

The research was carried out in four sites in England. This paper is concerned with the research that the author was involved with in the North‐East of England, in the town of Wallsend, a local authority area in North Tyneside. From the beginning of 2019 until lockdown in March 2020 a series of interviews, focus groups, notes from wanderings, attendance at local events, informal discussions with local people, and later, membership of a local writing group that generated a series of poems on Brexit (see website) made up the collection of fieldwork that this paper draws from. As noted above, there was a push in the early stages of the research to conduct the bulk of interviews and focus groups before ‘B‐day’ and indeed two of the focus groups took place on the 29 March 2019. In sum, five focus groups with 8–10 participants in each took place in early 2019 with an array of participants: a group of local people held in a stall at the indoor market, a group of local businesspeople organised via the Chamber of Commerce, a group of young adults from a local FE college, a group of people with supported Special Educational Needs and a group of young people at a local youth club. We also held small group conversations with approximately 30 older people at a local lunch club. Four in‐depth interviews took place with local people in leading positions in the community and without offering close identifiers these included: teachers, voluntary community organisers, and people active in local politics. Finally, two participants from the local FE college conducted photo diaries and two interviews themselves—this latter part of the project was curtailed under pandemic restrictions. The idea was to capture as wide a range of people and views as possible. People were not specifically asked about their orientation towards leave or remain but in most cases participants did clearly state their position as one or the other, or somewhere in between. In many senses, this voting position felt quite arbitrary to the discussions we were having because the complexities and contradictions in people's narratives did not map well onto these positions anyway as will be highlighted in the following discussion (see also Tyler and Blamire 2024). Instead, focus groups and interviews focused on people's grapplings with the future, typically for themselves, the country, their town and the people living there.

## 5 Wallsend: From Industrial to ‘Left Behind’

Any portrait of a place is inevitably incomplete. This one, for example, certainly does not resolve the need to see Wallsend as an area that is heterogeneous in its population, its political expression, and people's experience of, and proximity to poverty, and industrial decline. The aim here is to pull out key elements of its history that have led to the area, like many others, to come to be connoted with ‘left behindness’ and reveal how there is no clear or even path that leads to this identification. At the same time, the aim is to highlight the various ways through which the area has always struggled and had an intimate yet fluctuating relationship with the State and its imperial capitalist project, which in turn has meant that Wallsend has experienced a long unstable economic history.

Wallsend itself is a town situated just outside of Newcastle‐upon‐Tyne along the banks of the River Tyne. It gets its name because it is where Hadrian's wall ended and is the site of the Roman fort Segedunum. Historically, Wallsend colliery was a large provider of jobs. The pits closed in the 1930s but some of the local working men's clubs still draw attention to this link in their names. Wallsend is perhaps best known for its location and role as a key player in the shipbuilding industry (as well as being the birthplace of the musician, Sting). The imposing impact of the ships on the local community is viscerally captured in the work of local photographers like Chris Killip, with images familiar for capturing the seamless integration of ships lurching into terraced streets with children playing outside their houses in the foreground, apparently undisturbed by their presence. The Swan Hunter shipyard in Wallsend employed between 10 and 12,000 people at its height and built many well‐known ships including, the RMS Mauretania which was the fastest transatlantic crossing ship of its time and acted as a troop carrier and hospital ship during the Gallipoli campaign. The shipyard has thus facilitated a close imbrication between the area and the dynamics of global capitalism and acts a key symbol in the nation's story. However, this has not been a straightforward relationship and was always mediated by an erratic association with the State.

Johnman and Murphy's book (Johnman and Murphy 2002) details the relationship between British shipbuilding and the state and acts as a correction to what they describe as a widespread myth that the British shipbuilding industry has been a ‘bastion of laissez faire individualism’ on the part of owners and employers. Instead, they highlight how both shipbuilding and shipping have developed ‘hand and glove with the State’; a relationship that has been contained within various Acts of Parliament dating back to the late‐fourteenth century. These Acts, particularly from mid‐seventeenth century onwards, have cemented the association between the industry and the British imperial project. The Navigation Act of 1660, for example, restricted the transportation of colonial goods to ‘English’ ships on the pretext of England becoming ‘self‐sufficient’. However, whilst on the face of it this cementation of the industry's importance to the national project might imply it be rewarded with some sort of stability of structure and income for those areas where the industry dominated, this was not to be the case. Moreover, whilst shipyard closures in the twentieth century took place during economic downturns and thus appeared inevitable (largely occurring in two phases in the early and latter parts of the century, between 1909–1933 and 1960–1993), what becomes clear is that the decline of the industry in Britain was not solely linked to a decline in demand or uncontrollable global forces. Indeed, other European countries facing similar market forces fared better (Hobsbawm 1999; Johnman and Murphy 2002). Closures in the latter part of the twentieth century, have instead been linked to the ‘state‐sponsored annihilation of an industry’ from the late 1950s onwards (Johnman and Murphy 2002, 216) via the State's decision to privatise amidst a more stringent economic and ‘post’‐colonial context.

In the late 1970s the industry shifted from private sector to nationalised control as part of a rescue package following the oil crises and related economic downturn, and then returned to the private sector within a very short period of time before collapsing altogether (Johnman and Murphy 2002). This reiterated the unique status of this relatively precarious industry and its relationship to the State. From the 1980s onwards Britain ceased to be a ‘volume shipbuilder’ and levels of employment in the shipyards in Wallsend dropped significantly. Shipbuilding began to cease and construction facilities were closed entirely in 2007. Swan Hunter still exists locally but is limited to a small workforce with a focus on design and engineering. Hence, as with other heavy industries during the twentieth century, employment was less secure than is often implied by the narration of Britain's ‘great’ industrial past.

Wallsend has thus long been heavily impacted by the loss of industry, related job losses, fluctuating levels of employment and poverty. In amongst Chris Killip's collection, ‘The Last ships’^1^ and amongst those images of vast ships being constructed in the Tyne and leering into people's homes is a selection of images that lays bare the vastly unequal conditions of the shipbuilding industry. Photographs of poor‐quality housing in the midst of demolition and street walls and machinery emblazoned with graffiti urging locals not to vote in the 1975 referendum on the UK's European Communities membership viscerally offer up a counter narrative to that on which the current conjuncture relies. Since 2010, Wallsend has also been deeply impacted by austerity measures, which include impacts on local authority funding. In 2015, Wallsend was ranked within the top 20% of most deprived wards in England but by 2019 the local economy had worsened, and it is now ranked within the top 10% of most deprived wards (according to the Index of Multiple Deprivation). It sits in the region with the steepest increase of child poverty in the UK in the last decade, with 35% of babies, children and young people now living in poverty (Bailey 2024). Much of the work that is now available locally is unskilled and low paid, found in the service sector and is often on short term and unstable contracts. The council's regeneration ‘Masterplan’ published in 2010 set out ambitious plans for Wallsend, Willington Quay and the waterfront area. To date this regeneration has largely focused on the retail area in the town centre and has included a newly built library and renovations to The Forum shopping centre and indoor market, a new Aldi supermarket and related closures of smaller independent shops, a restoration project of the local park and the building of some ‘affordable’ housing.

### 5.1 What Is Left Behind?

When we situate local histories alongside the more recent story of left behindness the latter emerges even less sensibly as a useful and meaningful way to understand places like Wallsend. Wallsend's recognition as a place only recently hurt by global forces (inflicted internally and externally) is simply implausible and one that instead relies on a series of mythologies and rewritings of the English industrial landscape. The people we spoke to in Wallsend reaffirm this less than romantic image of stability, irrespective of where they stood in relation to the Brexit vote. Women reflected on how their husbands had been made redundant in the 1980s. And, we routinely heard reflections on the area such as:

When I left school in 1964 no one had a job.

It has always been that way.

[lack of jobs]

Things were shitty beforehand [before Brexit], it doesn’t make a difference if it is shitty after haha.

One woman when reflecting on Wallsend's past, in her response to a question about the future, posed the following question to me, ‘Do you remember Auf Wiedersehen Pet?’ By referring to a 1980s British sitcom, which depicted a group of unemployed construction workers from the North‐East of England forced to seek work in West Germany, she draws attention to the fragility of the local labour market but also the local knowledge that labour can, and often has to, come from multiple quarters, irrespective of borders. As such, local people are fully cognisant that their lives (especially via working men's lives) remain enmeshed within global markets, irrespective of their own orientations and feelings towards borders.

In one group, a comparison was made by a retired woman at the lunch club to rural parts of Italy where young people have left villages and entire regions to find work and sounded the death knell of small communities who were left without a future generation. And, whilst this comparison might seem on the face of it whimsical, it challenges the idea that Britain falls outside such comparisons with one of the poorest members of the EU. Indeed, there was a sense amongst several local business owners that Britain has ‘lost respect on global stage’ and that ‘Britain is a laughing stock’. What emerges as a recurring theme is that Britain as a whole has been thrown into flux at the hands of an elite with one man concluding, ‘It's Britain that has been left behind’ and another, ‘Britain is a hollow shell’.

Indeed, instability and uncertainty emerge as the only constants. And, whilst shipbuilding and related job sectors were not indicative of the zero‐hour contracts of today they did not provide the stability that is often implied. Work was beholden to the whims of the state, and competition for contracts. The imbrication of the State in Wallsend's ‘business’ is clearly felt. It was described by some members of a lunch club aimed at older people, that everything was and is ‘engineered for politicians’ interests' and to illustrate this they discussed a period in which contracts were redirected to Scotland. This period is detailed via the Union disputes between the yards in Wallsend and Govan in Scotland which ultimately saw the relocation of the work to Fairfields, Govan and the breaking of the strike. The union on Clydeside defending this decision via the need to prioritise local interests and in defence of national *British* interests (Phillips et al. 2021). Yet, many of the older participants reminisced on this period arguing that ‘*really*’ contracts and jobs were lost because the Labour government purposely redirected then to Scotland to appeal to voters.

Connected to this implicit knowledge was the conviction of many participants, including those who defined themselves as pro‐Brexit, that Brexit was being pushed because it would open a tax loophole for the wealthy. And again, irrespective of orientations towards Brexit and the racist discourses that cemented so much of the debates around it, there was a general sense that immigration was ‘all the talk of the time’ and acted as a distraction from these elite motivations. The orchestration of the various narratives that were produced alongside left‐behindness are not lost on the people of Wallsend. Indeed, the discussions about whether leave voters and more specifically white working class leave voters were ignorant/didn't know what they were voting for is a persistent yet reductive and unhelpful lens for understanding the current conjuncture but the accusation and indeed its repeated rebuttal does successfully divert attention from the collective nature of the discourses that underpinned the Leave campaign and its various articulations before and since.

In sum, the term ‘left behind’ is a slippery one that has a quality of its own. It simply alluded to the idea that some places are not faring as well, economically and socially, as they once were said to, or in comparison to cosmopolitan/metropolitan towns and neighbourhoods. One often cited critique of the ‘left behind’ claim is that the term is too easily connoted with pejorative *behindness*, insularity and parochialism (Mckenzie 2017b; Rodríguez‐Pose 2018). A case is made instead that it would be preferable to describe these towns as ‘left out’ to indicate their lack of political representation (Mckenzie 2017a). However, whilst these arguments have value—hard to dispute that the, implicitly white, working class is under‐represented and marginalised—they nevertheless reinforce a racialised class schism and somewhat miss the point. The very fact that ‘the left behind’ has been constructed by a broad political coalition to only identify English, white working‐class neighbourhoods (Local Trust 2023) cannot be a mistake. Does post‐industrial Port Talbot, or Ravenscraig, or the most diverse neighbourhoods in the west end of Newcastle not have as much in common with Wallsend in terms of levels of poverty, disenfranchisement and access to jobs and good housing? Why too is it that multicultural magnificent ports such as Butetown in Cardiff, are never referred to even as post‐industrial, and certainly not as ‘left behind’? Who, what and where can be defined as ‘left behind’ remains a subject under review. And so, whilst some remain steadfast about taking a pragmatic or instrumental approach to use this latest code for deprivation as a means to advocate for change (see e.g., MacKinnon et al. 2021; Rodríguez‐Pose 2018), we cannot ignore that the notion of being ‘left behind’ has been reserved within public and policy discourse for specific geographical areas and populations; those that are overwhelmingly white, provincial and have a working class and industrial heritage. Hence, we are inevitably left with the question as to what else is this term achieving and how is it useful.

## 6 The Sacrificial Quality of Nationalism and the Moral Imperative of Abandonment

This section examines how the ‘left behind’ has acted as a key vehicle for nationalism through its articulation with death through sacrifice, whilst also taking account of how this is contingent on the idea that some ‘thing’ is being taken from the nation, and the implications of this framing. In this way, it builds on the previous section by expanding upon the appeal of narratives of loss to nationalism. But it also pays particular attention to the parameters on which the nation is said to be under threat, who we should expect to accept sacrifice in this endeavour, and who we should abandon in the process.

‘Death!!’ Was the resounding cry from a young woman in a focus group in a FE college in response to the question, ‘What does Brexit mean for you?’ ‘I am more accepting about death now’, she goes on to explain, laughing. Studying for a social work qualification she described an anxiety about young people's futures, including her own. Delivered with some humour she nevertheless draws attention to a series of non‐futures that she and her peers detail in the form of the various cuts to services, the crisis in the NHS, the inadequacy of universal credit, benefit sanctions, the lack of jobs and housing available and the real fear or reality of homelessness for young people, as well as the closure of domestic violence shelters and support for young parents. There are, she simply states, ‘loads of things that can kill us’ now. Many older people empathised. They worried for their or the town's children and grandchildren facing ‘Zero hours contracts. 12 h 1 week and then 72 h the next. No holidays. No ability to plan their future’. They drew attention too to the hypocrisy and callousness of politicians with multiple houses making decisions about people's lives (not just their everyday lives but actual life itself) with an air of moral superiority.

In that same group, the young people discussed immigration at length, with attempts to evaluate the extent of the ‘problem’, whilst some argued it is not a problem at all. Several attempted to quantify ‘it’ in terms of how much was too much, and to determine whether the North‐East did have, as apparently one local councillor had informed a participant, a bigger problem than the rest of the UK, despite the North‐East being a much whiter than average region in England (93% compared to the national average of 81%, according to the 2021 Census). And equally, there were those young people who challenged many of these propositions and pointed instead to how many British *migrants* lived in Spain.

Unless you go and physically research it yourself, you only know what you are told in the media. And they are only going to tell you because they all want to steer you down a certain path with your thoughts.

These various nuances of, and resistances to, race remind us of the importance of not ‘resorting to deterministic conceptions of the relationship between race and class’ (Gilroy: 380). Nevertheless, they highlight how race and immigration relentlessly feature as an explanatory mechanism to make sense of the nation, even whilst it is understood as a device of distraction. These focus group conversations thus reflected what we might have come to see as a very typical discussion in a white English context and reflect the ways in which immigration has become a key explanatory tool for anything and everything and in such a way that relentlessly identifies migrants and racialised minorities as key threats to the nation.

There was a clear sense that Brexit would have no positive impact on Wallsend and that local people would continue to struggle and be called upon to make sacrifices in the process of ‘getting Brexit done’, as they had been under the regime of austerity. This is the case for participants irrespective of their Brexit perspective:

Unfortunately, it is the working‐class people in Wallsend who seem to be pulling in their belts tighter than most people, you know, you see houses in London going for ridiculous amounts of money and stuff like that.

[against Brexit].

The working classes have always taken the brunt of the pain and their pain has always been much harder. Like everything is much, much harder and harsher for the working classes.

[pro Brexit]

Where differences emerge then is in the orientation to the terms of the sacrifice. For some, it is not simply sacrifice for sacrifice's sake but rather a moral imperative, something dutifully given up for the nation. And through these narratives we see the already well‐embedded repertoires of English nationalism, that is that sacrifice is necessary to salvage the nation's integrity and industrial history, by protecting ‘our own’ and forsaking the lives of ‘others’ in the process. The following quote, for example, clearly outlines how the ‘pain’ that is described as inevitable from leaving the EU is nevertheless a sort of moral duty and investment for the nation's future.

Sometimes when people start squealing about a cut to living standards or not being able to have a foreign holiday or whatever, I just think ‘tough, get on with it’. It is kind of you know it is, not the end of the world… people yeah people have had to take pain. They have known their parents taking it, they have known their grandparents taking it, so therefore if we have to take, so that might be kind of our grandkids end up ultimately hopefully having a better life, then it is worth taking. Even if there is only a 50% chance that in after all of those time it does get better, then it is a risk worth taking…. I suppose it is like kind of the equivalent of an ISA.

But instead of putting money in you are putting pain in?

Yeah!

It is important to state that this conversation is also held in the shadow of her own orientations towards immigration and the contingent claim that racialised others should be abandoned. She states, for example, that the racist far right is not something she agrees with but rather ‘a means to an end’ to get Brexit done and ‘sovereignty’ restored. In this way she clearly states what some only hint at, that allegiance to the nation and its future simultaneously implies sacrifice (of ‘authentic’ national subjects) for the good of the nation, but also the need to forsake and abandon migrants and racialised ‘Others’ in the process. Indeed, she adds that their abandonment is necessary to make that sacrifice worthwhile drawing on the case of her son's girlfriend who, she concedes, would likely have to leave the country. In Wallsend, this dual logic of nationalism was indicated towards by participants with a diverse set of political affiliations, although there were clear divergences in their orientations towards the quality and extent of the claims underpinning this logic. For example, it was articulated amongst participants via explicitly racist Trump‐style demands to ‘build a wall’, as well as implicitly racist and more mainstream and politically diffuse demands to put ‘the British people’ first, or to redirect international aid money to protect ‘our own’ first or, ‘if big industries fail’. But it was also evident in the recognition, as noted above, that ‘the political world of *self‐interest*’, as one participant described it, meant that Brexit was employed as a means to further benefit the elite and ‘open a tax loophole for the wealthy’ at the expense of people in Wallsend yet, this was going unchecked because immigration had been purposely positioned as a distraction from this.

The imagined threat of immigration itself rarely speaks to anything closely tangible (because it cannot). Instead, conversations about migrants and their worth quite quickly give way to banal questions about identity and self‐sufficiency as an *identity*. Hence, within the discussions on immigration, participants veer towards questions such as this, succinctly posed by X:

The Brexit issue is really about or is partly about identity. What, where does Britain see itself and where does it want to go?

Even in rationalising their own adherence to racist immigration policies, the veneer of identity and the moral requirement to maintain its integrity is presented as such:

We are an island country. And kind of we should be able to secure our own borders, and make our own decisions…[DeGaulle] said, the thing about Island people is they're more, they're more open minded and they’re more adventurous because they live on an island. They also have a very strong identity. And that identity is protected by the water around their territory.

Indeed, for many participants, questions about identity and our need to salvage and protect ‘it’, which were repeatedly returned to, were closely bound up with the nation's ability to be self‐sufficient on ‘the island’. This is a discursive device that the Leave campaign relied upon heavily and speaks directly to the call to be racist and entrepreneurial at once in order to salvage the nation. It is hard not to see the traces and residues of the 1660 Navigation Act within the undercurrents of some of this speak. Brexit is, for example, described as a means to regain ‘sovereignty’, or control over both borders and the terms on which global trade is organised. Indeed, Labour's recent election campaign to support entrepreneurs and offer means to enable small business owner's to ‘take back control’ of the terms on which businesses can function is part of their pledge for a ‘patriotic economy’ (Lloyd 2024). In Wallsend, one local businesswoman helpfully illustrates this linkage very explicitly:

I would make the UK a tax haven and encourage entrepreneurial vision. We need to be part of the global world but reconnect on our own terms…we’ve been diminished, lost our sense of identity… It comes down to a defence of the realm… The realm must be defended at all costs. I would kind of, I would give my life for kind of senior members of the royal family. I would take a bullet for them. Deep down I’m a royalist. It is a DNA thing. It is in your blood. It is kind of, it is something that you can’t explain, you are born a royalist I don’t know, it is something in your DNA. It is passed down the line.

In thinking through the notions of immortality and fixity and their adherence to nationalism, Anderson (1983) proffers that the immortality of a nation is said to be guaranteed by the death of heroes fallen. Franklin (2019) expands on this same sort of thinking through what she calls the logic of sacrificial reproduction. This logic underpins the rationale on which sacrifices *have* to be made to guarantee the nation's future whilst also pointing to that which has been *taken* away and *should* be given back (Franklin 2019, 51). This also points to the extent to which nationalism acts as something more than a process of inclusion/exclusion (Valluvan 2019). The above discussion goes some way to illustrate how this works. The proposal here is that nationalism implies that the integrity of the nation relies on the forsaking or abandonment of racialised others and that this is distinct from, but connected to, the sacrifice expected of white English (working class) national subjects to fend off those encroaching alien threats.

## 7 Discussion

This paper has been concerned with how nationalism is convened and condensed in this moment by exploring the function of notions of loss and death in its articulation. It has worked with the proposition that death possesses an ideological currency; an alluring quality that equips nationalism with a moral imperative. The thinking behind this paper came about from trying to make sense of, on the one hand, the narratives of people living in a post‐industrial town in the North‐East of England and how they envisaged (or not) futures after Brexit, whilst simultaneously trying to make sense of how these very same places which became labelled as ‘left behind’ became somewhat untimely symbols of nationalist demands and expectations. What becomes apparent from even a cursory examination of the ‘Brexit’ rhetoric is the ways in which the past (and what is said to have been lost) is so heavily relied upon as a means to reconfigure the future. What I have tried to do here is explicate a series of contradictory narratives that surface in the telling of local histories that are deeply embedded in the wider context of industrialisation and deindustrialisation. In this way tensions between dominant representations of ‘great’ British industry and suppressed residual local histories that disturb this view can be identified. This can then help us understand the function of nationalism in the current conjuncture, and, why the cipher of the ‘left behind’ and industrial decline has become such a useful one to nationalism's purpose.

People in Wallsend from across the political spectrum identified the various sacrifices that they and, in particular, young people are expected to make even whilst their own orientations towards those sacrifices may differ. And, simultaneously they also identify how migrants and racialised ‘others’ are forsaken and abandoned in the same process. This is an important distinction to make. All too often the debate about who comes to matter descends quickly into manufactured racialised schisms that privilege whiteness in times of economic hardship. The proposal here then has thus been that nationalism implies that the integrity of the nation relies on the forsaking or abandonment of racialised others and that this is distinct from, but connected to, the sacrifice expected of white English (working class) subjects to fend off encroaching global forces.

The paper has illustrated how the left behind has become a useful axiom precisely because it can do a great deal of work without inhibiting nationalist triumphalism. And so, whilst there may be a concession that policy makers have forgotten these places and like elsewhere in the UK, they are suffering from dire levels of poverty, it is the very fact that they are predominantly white and are no longer industrial powerhouses that becomes significant and of national import. The ‘shreds and patches’ (Gellner 1983) of industry are cast together to form an imaginary of a lost triumph that has been ‘hurt by globalisation’. Nationalism permits liability to lay at the feet of those encroaching global forces via internal and external borders, but does not allow for introspection or re‐examination of national social and economic reforms. Crucially then, the ‘left behind’ in Wallsend and elsewhere become the symbol of the nation's fallen that ‘we’ must now collectively resurrect but they are also cast as symbolic of the perennial threat from alien forces. The distinction between requisites of sacrifice and abandonment hopefully goes some way to resolve some of the tensions that are present in some discussions of the race‐class relationship. To put it crudely, white working classes are called upon to make sacrifice in the name of the nation, whilst racialised minorities are abandoned in that same process. The discussion has highlighted how this is appealed to via the need for entrepreneurial self‐preservation, which aligns with the neoliberal project, but has its roots in colonial enterprise and thus emerges particular pertinently in the context of deindustrialisation and the narrated ‘death’ of communities that were integral to Britain's imperial ambitions.

In many ways, Wallsend and its ‘left‐behind’ counterparts merely act as props in the signification of loss and its centrality to nationalism. The articulation of industry with death of community and nation via the motif of the ‘left behind’ opens up spaces to grieve the loss of industry without acknowledging how that loss was made contingent. By examining narratives of nationalism that have taken hold in the current conjuncture but positioning these within a longer history I hope to have explicated how a post‐industrial melancholia, which cannot be disentangled from its postcolonial lineage, acts to reconfigure the past and reconcile the nation's demise. In this way ‘the left behind’ can be cast as the latest victims of global forces and as symbolic of the wider risk to the nation. The narrative of loss as a claim is often successful simply because people have lost jobs, but the signification of the left behind is more than this. It indicates a post‐industrial melancholic space in which we can grieve the loss of England's position in the world and relationship to global capitalism and a place on the ‘world stage’ (whatever that means) and transfers a moral conviction to narratives of nation that infer how lives matter and how they do not.

## Conflicts of Interest

The author declares no conflicts of interest.

## Data Availability

The author has nothing to report.

## Endnotes

^1^

https://laingartgallery.org.uk/whats‐on/chris‐killip‐the‐last‐ships.
